# The First Genome Survey and De Novo Assembly of the Short Mackerel (*Rastrelliger brachysoma*) and Indian Mackerel (*Rastrelliger kanagurta*)

**DOI:** 10.3390/ani12141769

**Published:** 2022-07-10

**Authors:** Komwit Surachat, Patcharaporn Narkthewan, Chayanin Thotsagotphairee, Monwadee Wonglapsuwan, Walaiporn Thongpradub

**Affiliations:** 1 Department of Biomedical Science and Biomedical Engineering, Faculty of Medicine, Prince of Songkla University, Hat Yai, Songkhla 90110, Thailand; komwit.s@psu.ac.th; 2 Molecular Evolution and Computational Biology Research Unit, Faculty of Science, Prince of Songkla University, Hat Yai, Songkhla 90110, Thailand; 3 Food and Agricultural Biotechnology Program, Department of General Science and Liberal Arts, King Mongkut’s Institute of Technology Ladkrabang Prince of Chumphon Campus, Pathiu, Chumphon 86160, Thailand; patcharaporn.na@kmitl.ac.th; 4 Science Education Program, Department of General Science and Liberal Arts, King Mongkut’s Institute of Technology Ladkrabang Prince of Chumphon Campus, Pathiu, Chumphon 86160, Thailand; thotsagotphairee@gmail.com; 5 Division of Biological Science, Faculty of Science, Prince of Songkla University, Hat Yai, Songkhla 90110, Thailand; 6 Center for Genomics and Bioinformatics Research, Faculty of Science, Prince of Songkla University, Hat Yai, Songkhla 90110, Thailand

**Keywords:** short mackerel, Indian mackerel, *Rastrelliger brachysoma*, *Rastrelliger kanagurta*, genome survey, whole-genome sequencing, de novo assembly

## Abstract

**Simple Summary:**

Mackerel species are commercially important marine species in Southeast Asia, especially short mackerel and Indian mackerel. However, genomic information about them is still limited. Genome survey of these two mackerel species was reported in this study. Next-generation sequencing and comprehensive bioinformatics were performed to obtain the genetic information. The estimated genome size of both species is around 680 Mbp. The heterozygosity of these species was very similar, while the repeat content for Indian mackerel was slightly higher than for short mackerel. Functional annotation also was reported in this study. This is the first reported genome survey and assembly of species in the genus *Rastrelliger* and could be useful for future comparative genomic studies.

**Abstract:**

*Rastrelliger brachysoma* (short mackerel) and *Rastrelliger kanagurta* (Indian mackerel) are commercially important marine species in Southeast Asia. In recent years, numbers of these two species have been decreasing in the wild, and genomic information about them is still limited. We conducted a genome survey of these two mackerel species to acquire essential genomic information using next-generation sequencing data. To obtain this genetic information, comprehensive bioinformatics analyses were performed, including de novo assembly, gene prediction, functional annotation, and phylogenetic analysis. The estimated genome sizes were around 680.14 Mbp (*R. brachysoma*) and 688.82 Mbp (*R. kanagurta*). The heterozygosity of these species was very similar (≈0.81), while the repeat content for *R. kanagurta* (9.30%) was slightly higher than for *R. brachysoma* (8.30%). Functional annotation indicated that most of the genes predicted in these two species shared very close average amino acid identities (94.06%). The phylogenetic analysis revealed close phylogenetic relationships between these two species and other scombrids. This is the first reported genome survey and assembly of species in the genus *Rastrelliger* and could be useful for future comparative genomic studies.

## 1. Introduction

The *Rastrelliger* genus belongs to the family Scombridae, which includes epipelagic fishes found in tropical and subtropical regions. There are three species in the *Rastrelliger* genus: *Rastrelliger brachysoma* (short mackerel), *Rastrelliger kanagurta* (Indian mackerel), and *Rastrelliger faughni* (island mackerel). The short mackerel and Indian mackerel are economically important species in the Gulf of Thailand. However, the numbers of both species are seriously declining in the wake of large annual captures [1,2]. The fishery and aquaculture statistics published by the Food and Agriculture Organization of the United Nations (FAO) revealed that *R. kanagurta* and *R. brachysoma* were among the 70 principal species with capture productions of more than 150,000 tons in 2019 [3].

*R. brachysoma* has a relatively shallow body and its head length is equal to or less than its body depth. Its dorsal fin is yellowish with a black rim. The caudal fin is also yellowish, whereas the pectoral and pelvic fins are dusky [4,5]. *R. brachysoma* is widespread in Southeast Asia. The distribution of this species in the Central Indo-West Pacific extends from the Andaman Sea eastward to Fiji and from Indonesia northward to the northern Philippines. *R. brachysoma* is found in estuarine habitats and offshore areas where sea surface temperatures range between 20 °C and 30 °C [4]. *R. kanagurta* has a moderately deep body but its body depth is shorter than its head length. This species has narrow dark longitudinal stripes along the upper part of the body and black spots on the body near the lower margin of the pectoral fin. The pectoral, caudal, and dorsal fins are yellowish [4,5]. *R. kanagurta* is found in the Indo-West Pacific from South Africa and the Red Sea eastward to the Samoan Islands and from the north coast of Australia northward to southern Japan. It is found in shallow waters where the sea surface temperature is around 17 °C [4].

The development of next-generation sequencing (NGS) technology has accelerated the investigation of genome structure, gene expression, and gene control. Whole genomes of laboratory model fish, such as zebrafish and medaka, have now been reported, and several biomarkers have been developed. Where the family Scombridae is concerned, the investigation of whole genome sequences has been confined to fish of high commercial value, such as the Pacific bluefin tuna (*Thunnus orientalis*), the southern bluefin tuna (*Thunnus maccoyii*), and the yellowfin tuna (*Thunnus albacares*). Hence, although the *Rastrelliger* genus is commercially important in countries around the Gulf of Thailand, none of the genomes from any of the species of the genus have yet been reported.

Therefore, this study aimed to conduct a genome survey of the *Rastrelliger* genus along with a comparative genomics analysis to determine the evolutionary links to related species. We focused on *R. brachysoma* and *R. kanagurta* since they are economically important species in Thailand. This genome survey provides information about genome size, genome content, and phylogenetic relationships among fish in the same family. The information acquired could benefit future studies of the molecular mechanisms of these species, especially reproductive mechanisms, and improve the accuracy of transcriptome data. A better understanding of their reproductive mechanisms could enable the farming of these two fishes, which is not yet feasible. This information also has relevance for future studies of related species. To further our understanding of the relevant molecular mechanisms, the complete genomes and transcriptomes of these two species will eventually be investigated.

## 2. Materials and Methods

### 2.1. Sample Collection

**Figure 1:**
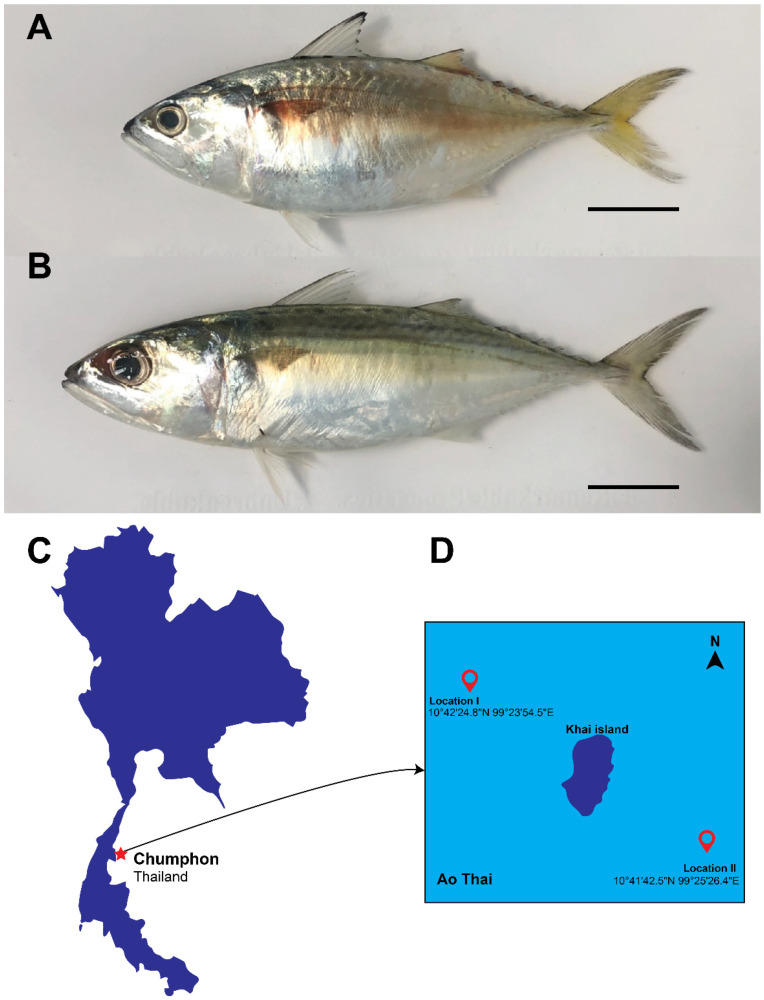
Images of *R. brachysoma* (**A**) and *R. kanagurta* (**B**) from Ao Thai, Chumphon Province, Thailand. Scale bars represent 3 cm. (**C**) Chumphon Province (red star). (**D**) Sampling site location.

One specimen of *R. brachysoma* and one of *R. kanagurta* were obtained from Khai Island, Chumphon Province, Thailand (Figure 1). This study was performed under the guidelines of the Animal Care and Use Committee of King Mongkut’s Institute of Technology Ladkrabang, Thailand (approval no. ACUC-KMITL-RES/2022/003).

### 2.2. DNA Extraction and Genome Sequencing

Genomic DNA was extracted from the caudal fin of each sample using a Gentra^®^ Puregene^®^ Kit (QIAGEN, MD, USA) according to the manufacturer’s protocol. Briefly, we first incubated fin tissue (20 mg) with 600 µL of cell lysis solution and 60 µg of proteinase K at 55 °C overnight. The next day, we added 12 µg of RNase A and continued incubation for 30 min at 37 °C. We then added 200 µL of protein precipitation solution, precipitated the proteins for 15 min in ice, and centrifuged the tubes at 13,200 rpm for 15 min at 4 °C. The supernatant was transferred to a new tube, 600 µL of cold isopropanol was added, and the tube was centrifuged again in the same condition to precipitate DNA. The obtained pellet of genomic DNA was washed in 70% ethanol and spun at 13,200 rpm for 10 min at 4 °C. The DNA was resuspended in DI water and stored at −20 °C until used.

Two genomic DNA libraries of the samples were built using a TruSeq Nano DNA Kit (San Diego, CA, USA) following the manufacturer’s instructions. The insert size of the prepared libraries was 350 bp. The sequencing process was then initiated with an Illumina NovaSeq 6000 sequencing system from Macrogen (Seoul, Korea). The reads generated on the sequencer were 2 × 150 bp paired-end reads.

### 2.3. Genome Size Estimation

The raw reads from both sequencing libraries were cleaned and low-quality data filtered using Trimmomatic v0.32 [6] with default parameters (ILLUMINACLIP: TruSeq3-PE.fa:2:30:10 LEADING: 3 TRAILING: 3 SLIDINGWINDOW: 4:15 MINLEN: 36). The cleaned reads were then used in k-mer analysis to estimate the genome size. To determine k-mer frequency from the input sequence data, jellyfish v2.3.0 [7] was used with a k-mer length of 21. The histograms were then exported from the k-mer counts. The genome heterozygosity, repeat contents, and size of the *R. brachysoma* and *R. kanagurta* samples were then estimated with GenomeScope webserver v1.0 [8] using a kmer-based statistical approach.

### 2.4. De Novo Genome Assembly and Gene Prediction

To assemble the *R. brachysoma* and *R. kanagurta* genomes, we applied a previously described method [9]. Briefly, SOAPdenovo2 [10] was used to build a de novo draft assembly with a k-mer size of 41. The gaps that emerged during the scaffolding process with the assembled results were closed with GapCloser v1.12 [10]. The quality assessment of genome assemblies was evaluated using QUAST v5.1.0 [11], generating metrics based only on scaffolds results. Benchmarking Universal Single-Copy Orthologs (BUSCO) v5.3.2 [12] was used to check genome completeness and to analyze both genomes in the vertebrata_odb10 database. Gene predictions were then performed with AUGUSTUS v3.4.0 [13] using default parameters and setting zebrafish as the gene model. The functional annotations were predicted using eggNOG-mapper v2.0 [14,15] against the KOG, KEGG, GO, and PFAM databases.

### 2.5. Genome Similarities with Other Species and Phylogenetic Analysis

The genome similarities between *R. brachysoma* and *R. kanagurta* and other species were determined with NCBI megablast [16] using several scaffolds for both fishes. Species closely related to the two species of interest were then selected for use in the phylogenetic analysis. A total of 13 fish species (Table S1) were included in the analysis, using *Danio rerio* as an outgroup. Single-copy ortholog genes were extracted with BUSCO from the annotation files of the selected species, using vertebrata_odb10 as a target database. The extracted single-copy ortholog genes of all 13 samples were aligned with MUSCLE v5.1 [17]. A maximum-likelihood (ML) phylogenetic tree was constructed based on GTR and GAMMA correction using raxmlHPC-PTHREADS v8.211 [18], setting *Dario rerio* as an outgroup. The bootstrap values were calculated using 1000 replicates.

### 2.6. Identification of Microsatellite Motifs in Fish Genomes

Simple sequence repeats (SSR) or microsatellites in the genomes were identified with a MISA Perl script [19]. The parameters were set for the identification of mono-, di-, tri-, tetra-, penta-, and hexa-nucleotide microsatellite motifs with a minimum of 10, 6, 5, 5, 5, and 5 repeats, respectively.

### 2.7. Mitochondrial Genome Assembly

To identify the mitochondrial genome sequences, the high coverage contigs were identified by running the assembly results for both genomes through MitoZ v2.4 [20] with the findmitoscaf module. The annotations and visualizations of the mitochondrial genomes were also generated using the annotate and visualize modules of MitoZ, respectively. The obtained mitochondrial genome sequences of both fishes were then compared with sequences of other related species downloaded from the National Center for Biotechnology Information (NCBI) database. Sequences of 13 protein-coding genes (PCGs) and two rRNA sequences were extracted from each genome and concatenated to perform multiple sequence alignment (MSA) and construct a phylogenetic tree. Sequences were then aligned using MUSCLE v5.1 [17]. A maximum-likelihood (ML) phylogenetic tree was constructed based on GTR and GAMMA correction with 1000 bootstrap values using raxmlHPC-PTHREADS v8.211 [18]. In this tree, the zebrafish was used as an outgroup.

## 3. Results and Discussion

### 3.1. Genome Sequencing Statistics and Genome Size Estimation

**Figure 2:**
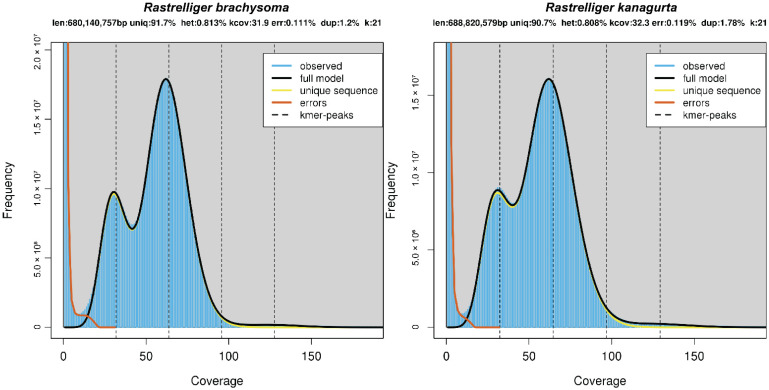
21-mer analysis for estimation of the genome size of *R. brachysoma* and *R. kanagurta*.

The raw data for *R. brachysoma* and *R. kanagurta* obtained from the sequencer had total sizes of 73.4 and 77.2 Gbp, respectively. After the raw data were cleaned, the total file sizes of both read libraries were 68.9 and 71.4 Gbp, respectively. The genome sizes of the two specimens were estimated from k-mer analyses, which showed that the peak 21-mer distributions of both were at 62× (Figure 2). The estimated genome sizes of *R. brachysoma* and *R. kanagurta* were 680.14 Mbp and 688.82 Mbp, respectively. The heterozygosity and repeat content information are given in Table 1.

Roughly speaking, the genome sizes of *R. brachysoma* and *R. kanagurta* are 680–700 Mbp, and the genome of *R. brachysoma* is slightly smaller than that of *R. kanagurta*. Both genomes are smaller than those of some other fish in the family Scombridae. For example, the Pacific bluefin tuna (*Thunnus orientalis*), the southern bluefin tuna (*Thunnus maccoyii*), and the yellowfin tuna (*Thunnus albacares*) have genomes of 787 Mbp [21,22], 782 Mbp, and 836 Mbp [23,24], respectively. Nonetheless, both genomes are bigger than the genome of the Atlantic bluefin tuna (*Thunnus thynnus*), which is 648 Mbp. The heterozygosity index of *R. brachysoma* (0.813) is slightly higher than that of *R. kanagurta* (0.808). Some years ago, Adelyna et al. [25] performed a genome survey and identified microsatellite markers in *R. brachysoma* using the Ion Torrent sequencing platform. The results provided only a partial genome survey since the low-depth sequencing coverage of 81.28 Mbp enabled them to assemble only short contigs. In this study, we used a higher resolution sequencing depth to provide more information about the two species. The resulting higher throughputs and increased data enabled functional annotation, gene prediction, and microsatellite investigation.

### 3.2. Genome Assembly and Gene Prediction

The genome assemblies of *R.* *brachysoma* and *R. kanagurta* were undertaken by varying k-mer values from 21 to 63 to generate the best draft assembly. A total of 213,093 and 292,418 scaffolds were generated from *R. brachysoma* and *R. kanagurta* with N50 values of 4198 and 2681, respectively. The completeness of the genome assemblies of these two fishes was 30.7% for *R. brachysoma* and 22.8% for *R. kanagurta* (Figure S1). The assembly statistics are illustrated in Table 2.

**Figure 3:**
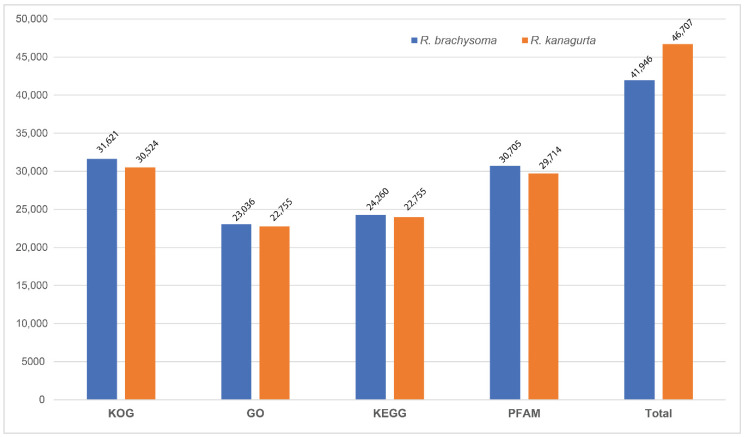
Functional annotation statistics for *R. brachysoma* and *R. kanagurta*.

Only short-read sequencing was used to assemble genomes in this study. Therefore, it was quite difficult to obtain good assembly results, since the repeat content within genomes could interfere in the assembly process. For instance, the genome of *R. kanagurta* contained a higher ratio of repeated sequences than *R. brachysoma*, which directly affected the N50 value and number of scaffolds. Gene prediction by AUGUSTUS software produced totals of 41,946 and 46,708 genes for *R. brachysoma* and *R. kanagurta*, respectively. These predictions were slightly lower than the gene predictions for *T. maccoyii* (49,507) and *T. albacares* (48,150), which are also from the family Scombridae. For both *R. brachysoma* and *R. kanagurta*, the number of genes per genome was high in comparison to other organisms, such as the Hereford cow (~22,000) [26] and white shark (~24,520 genes) [27]. It is possible that the ancestor of these two species underwent the 3R, or fish-specific genome duplication, event. Several studies have reported the importance of teleost-specific whole-genome duplication (TS-WGD) for the development of teleost complexity [28,29]. Duplication of the genome might explain the very high gene numbers found in the present study. Functional annotations were assigned to predicted genes using the KOG, GO, KEGG, and PFAM databases (Figure 3). Over 65% of all predicted genes in both species were assigned in at least one database, while the others could not be assigned to any database because of the sequence length and N values within the protein sequences.

**Figure 4:**
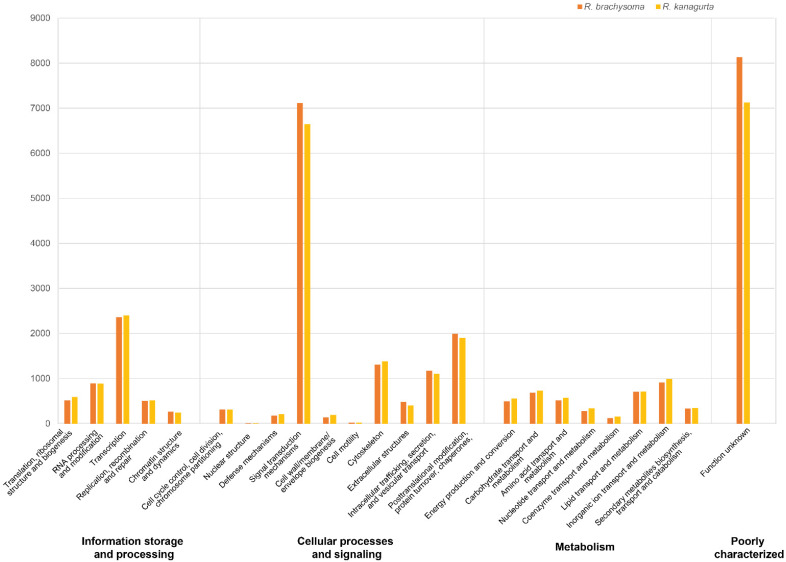
Classification of Eukaryotic Orthologous Groups (KOGs) in gene prediction. Orange bars represent the KOGs of *R. brachysoma* and yellow bars the KOGs of *R. kanagurta*. KOG categories appear on the horizontal axis, and the frequencies of the categories are indicated along the vertical axis.

For KOG annotation, the highest frequency (approximately 23%) predicted by eggNOG-mapper for both samples was for “function unknown” (S category) (Figure 4). The second highest frequency (around 20%) was for the signal transduction and mechanisms categories. The other categories of these two species were very close. This result could indicate that most of the predicted genes in *R. brachysoma* and *R. kanagurta* share very close functional coding sequences and phylogenetic relationships. To provide better assembly and annotation results, mate-pair and third-generation sequencing—e.g., Oxford Nanopore and PacBio—could provide longer DNA sequences that are simpler to assemble into longer sequences and easier to use for annotation.

### 3.3. Genome Similarities with Other Species and Phylogenetic Analysis

The average similarity between *R. brachysoma* and *R. kanagurta* scaffolds and other species was determined from the NCBI database using BLASTn and average amino acid identity was determined from the protein orthologs using BLASTp. The results are given in Table 3. The similarity search for *R. brachysoma* and *R. kanagurta* showed that identity and coverage taken together were closest to *T. albacares* and *T. maccoyii*, which was not surprising since all four species are from the Scombridae family. The other BLASTn matches, with coverage from 42–45%, were from different species: *Anabas testudineus*, *Lateolabrax maculatus,* and *Epinephelus lanceolatus*, for example.

The BLASTn results were consistent with the BLASTp results and returned similar fish species (Table 3). Only *L. maculatus* did not generate a hit; however, this is not an extraordinary result since only 613 proteins of the species have been deposited in the NCBI database. The amino acid identity results for the protein orthologs were close to the nucleotide identity, while their coverage percentages ranged from 95–100%. However, the coverage percentages of the BLASTn search were only around 50–60, which indicated a degree of genome differentiation among these species and that higher resolution sequencing should be conducted in the future to provide more information on these species.

**Figure 5:**
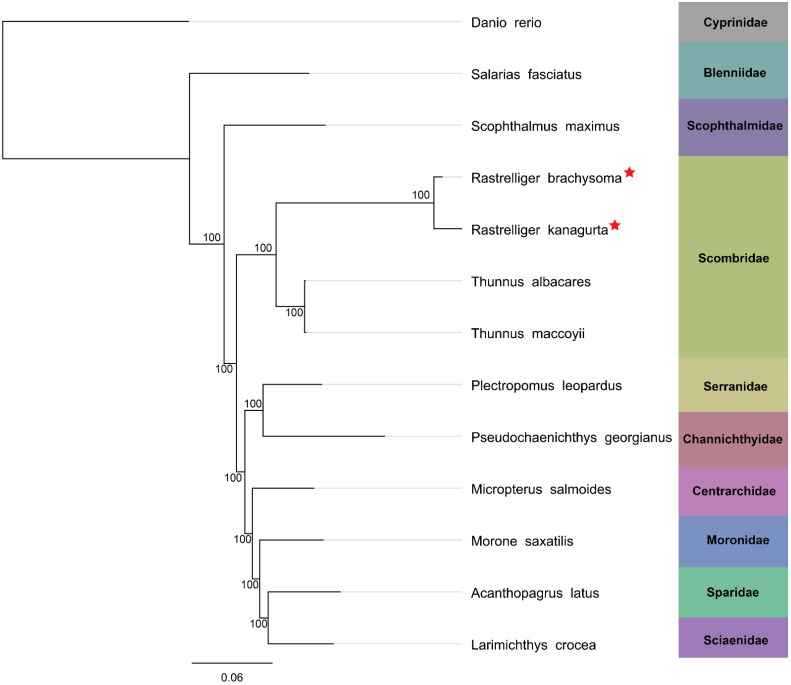
The phylogenetic tree was inferred from 305 single-copy ortholog genes from 13 species using the neighbor-joining method with 1000 bootstrap replicates in the Geneious software, setting zebrafish as an outgroup. The red star represents the species used in this study.

The annotation files of 13 other fish species were downloaded from the blast hit results to construct the phylogenetic tree. Only shared single-copy ortholog genes were used to build the tree. These included 305 genes. As a result, *R. brachysoma* and *R. kanagurta* formed the same clade with two species from the genus *Thunnus*, which were also from the same family (Figure 5). In addition, each species included in the analysis formed a distinct cluster based on their family. This result was consistent with the blast identification, which showed a close relationship between *R. brachysoma*, *R. kanagurta*, *T. albacares*, and *T. maccoyii* and other species in other families; e.g., Serranidae and Moronidae.

### 3.4. Identification of SSRs

In the present study, a total of 274,764 and 273,175 SSRs, respectively, were identified by MISA script based on the draft genome assemblies of *R.* *brachysoma* and *R. kanagurta*. The frequencies of SSR motifs identified in both genomes are provided in Table 4. The distribution frequencies of the microsatellites of these two species were very close at approximately 400 microsatellites per million base pairs. The choice of simple sequence repeats (SSRs) as genetic markers in this genome survey was made after a review of the relevant literature. SSRs have been used as genetic tools in fish and aquaculture for linkage map construction [30], assessment of genetic diversity [31], parentage determination [32], and a genome-wide association study [33]. The information on SSRs in *R.* *brachysoma* and *R. kanagurta* could be benefit further studies of genetic markers in mackerels.

### 3.5. Mitochondrial Genome Assembly and Comparative Analysis

**Figure 6:**
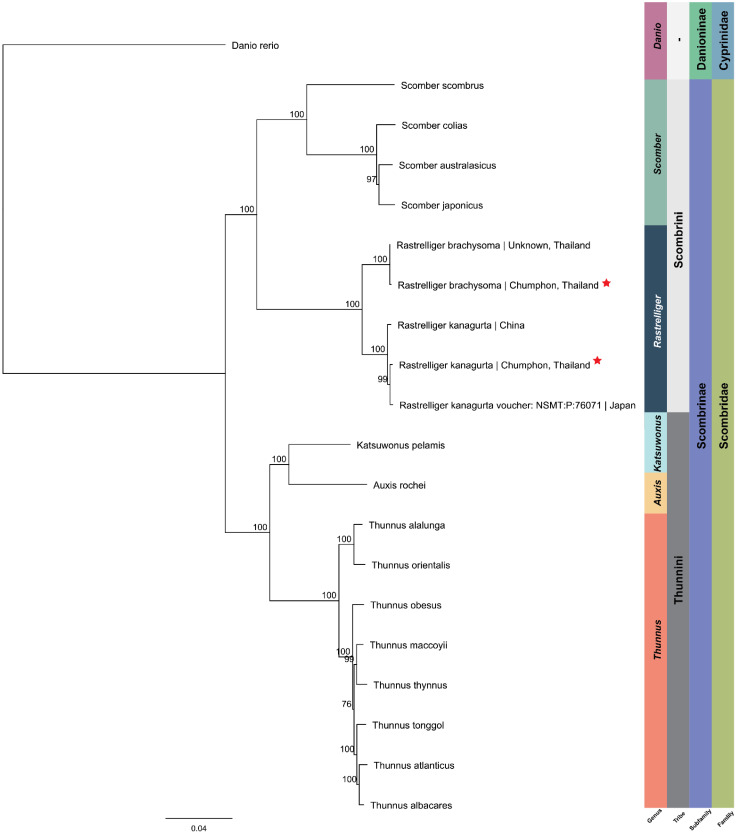
The phylogenetic tree was constructed from mitochondrial genomes of 20 species from the family Scombridae using zebrafish as an outgroup. The red star represents the species used in this study.

The complete mtDNA genomes of *R. brachysoma* and *R. kanagurta* were circular molecules with total lengths of 16,539 and 16,537, respectively. The mtDNA sequences of both species comprised 22 tRNA, 2 rRNA, and 13 protein-coding genes. These two mtDNA genomes shared 96.64% similarity, while the GC content of *R. kanagurta* (47.7%) was slightly higher than that of *R. brachysoma* (47.5%). We then used these two sequences to construct a phylogenetic tree with another 16 species from the family Scombridae and one species from the family Cyprinidae (*Danio rerio*) (Table 5 and Figure 6).

Not surprisingly, the tree was clustered by family, subfamily, tribe, and genus, respectively. All complete mtDNA sequences of related species of *R. brachysoma* and *R. kanagurta* were selected from the NCBI database to construct the tree. However, the mtDNA sequence of the island mackerel (*Rastrelliger* *faughni*) was not yet complete at the time of the study and only DNA fragments from some genes (e.g., *cytB*, *COX1*) were present in the database. Moreover, *R. brachysoma* shared 99.9% similarities between this study and a previous study [34]. Only 10 single-nucleotide polymorphisms (SNPs) were found in the protein-coding genes. These two fishes were both caught in Thai waters, one from the south of Thailand and one from an unknown location. In addition, *R. kanagurta* shared 99.49% and 99.71% similarities with the same fish species from China [35] and Japan [36], respectively. We completed a phylogenetic analysis of species from the family Scombridae. The results showed that the phylogenetic relationships of each species were explicitly clustered into individual clades. The genetic distances of each fish in the same genus were very small but these data can still be used to clarify the taxonomic classification of these species.

## 4. Conclusions

The first genome assembly of *R. brachysoma* and *R. kanagurta* was investigated, and a genome survey and genomic insights into the genetic contents of these two fishes were provided. To our knowledge, this is also the first whole-genome sequence report for species from the genus *Rastrelliger*. The estimated genome sizes of *R. brachysoma* and *R. kanagurta* were smaller than those of species from the genus *Thunnus*, which is also in the family Scombridae. While the sizes and numbers of predicted genes were slightly different between *R. brachysoma* and *R. kanagurta*, the phylogenetic analysis, based on single-copy ortholog genes and mtDNA sequences, showed close phylogenetic relationships between these two species and *Thunnus albacares* and *Thunnus maccoyii*. This study could provide important genetic and phylogenetic information about *Rastrelliger* for further research. However, high-resolution and long-read sequencing should be conducted to improve the assemblies and annotation of these two draft genomes.

## Figures and Tables

**Table 1:** Genome size estimation statistics based on 21-mer analysis of *R. brachysoma* and *R. kanagurta*.

Species	k-mer	k-mer Depth	Estimated Genome Size (Mbp)	Heterozygous Ratio (%)	Repeat Ratio (%)
*R. brachysoma*	21	62	680.14	0.813	8.30
*R. kanagurta*	21	62	688.82	0.808	9.30

**Table 2:** Genome assembly statistics for *R. brachysoma* and *R. kanagurta*.

Species	Results	Length (bp)	Total Number	Max Length (bp)	N50	L50
*R. brachysoma*	contig	1,470,475,468	344,536	8220	907	111,256
	scaffold	562,970,690	213,093	107,797	4198	29,761
*R. kanagurta*	contig	1,403,153,623	499,510	4142	701	76,797
	scaffold	548,629,566	292,418	44,811	2681	55,832

**Table 3:** The average nucleotide and amino acid similarity between *R. brachysoma* and *R. kanagurta* and other species.

**Family**	**Species**	**Nucleotide Level**			
		***R. brachysoma***		***R. kanagurta***	
		**Identity (%)**	**Coverage (%)**	**Identity (%)**	**Coverage (%)**
Scombridae	*T. albacares*	86.55	58.00	85.47	56.00
Scombridae	*T. maccoyii*	86.49	57.00	85.52	54.00
Anabantidae	*A. testudineus*	82.22	45.00	84.88	40.00
Lateolabracidae	*L. maculatus*	82.02	42.00	88.29	42.00
Serranidae	*E. lanceolatus*	80.99	47.00	87.63	44.00
**Family**	**Species**	**Amino Acid Level**			
		***R. brachysoma***		***R. kanagurta***	
		**Identity (%)**	**Coverage (%)**	**Identity (%)**	**Coverage (%)**
Scombridae	*T. albacares*	89.99	99.4	86.53	98.25
Scombridae	*T. maccoyii*	89.94	99.4	88.78	97.40
Anabantidae	*A. testudineus*	81.45	99.4	83.03	97.60
Lateolabracidae	*L. maculatus*	NA	NA	NA	NA
Serranidae	*E. lanceolatus*	83.59	95.60	84.60	97.00

**Table 4:** Frequency of SSR motifs identified in *R. brachysoma* and *R. kanagurta*.

Species	Total	Di-	Tri-	Tetra-	Penta-	Hexa-
*R. brachysoma*	274,764	216,353	34,265	21,544	2350	252
*R. kanagurta*	273,175	222,343	33,950	14,992	1811	79

**Table 5:** mtDNA sequences used in phylogenetic analysis.

No	Fish Name	Species	Location	Number of bp	Accession No.	Reference
1.	Short mackerel	*Rastrelliger brachysoma*	Thailand	16,539	OM460828	This study
2.	Short mackerel	*Rastrelliger brachysoma*	Thailand	16,539	EU555283	[34]
3.	Indian mackerel	*Rastrelliger kanagurta*	China	16,537	JX524134	[35]
4.	Indian mackerel	*Rastrelliger kanagurta*	Thailand	16,537	OM460829	This study
5.	Indian mackerel	*Rastrelliger kanagurta*	Japan	16,537	AP012948	[36]
6.	Blue mackerel	*Scomber australasicus*	Japan	16,570	AB102725	-
7.	Atlantic chub mackerel	*Scomber colias*	Spain	16,570	AB488406	[37]
8.	Chub mackerel	*Scomber japonicus*	Japan	16,568	AB102724	-
9.	Atlantic mackerel	*Scomber scombrus*	Unknown	16,558	MN122853	-
10.	Bullet tuna	*Auxis rochei*	Philippines	16,505	MW232421	-
11.	Skipjack tuna	*Katsuwonus pelamis*	Philippines	16,514	MW232429	-
12.	Longfin tuna	*Thunnus alalonga*	China	16,527	KP259549	[38]
13.	Yellowfin tuna	*Thunnus albacares*	China	16,528	KP259550	[39]
14.	Bluefin tuna	*Thunnus orientalis*	Japan	16,527	GU256524	-
15.	Atlantic bluefin tuna	*Thunnus thynnus*	Japan	16,527	GU256522	-
16.	Southern bluefin tuna	*Thunnus maccoyii*	Japan	16,527	JN086150	-
17.	Bigeye tuna	*Thunnus obesus*	Japan	16,528	JN086152	-
18.	Blackfin tuna	*Thunnus atlanticus*	Mexico	16,528	KU955343	-
19.	Longtail tuna	*Thunnus tonggol*	Philippines	16,529	MW232430	-
20.	Zebrafish	*Danio rerio*	Unknown	16,596	AC024175	-

## Data Availability

The short mackerel and Indian mackerel genome projects were registered in NCBI under Bioproject numbers PRJNA850182 and PRJNA850176, respectively.

## Supplementary Materials

The following supporting information can be downloaded at: https://www.mdpi.com/article/10.3390/ani12141769/s1, Figure S1: BUSCO completeness assessment of *R. brachysoma* and *R. kanagurta*, Table S1: Whole-genome sequences of 20 fish species used in the maximum-likelihood (ML) tree construction.

## References

[1] Kongseng S., Phoonsawat R., Wanchana W., Swatdipong A. (2021). Genetic mixed-stock analysis of short mackerel, *Rastrelliger brachysoma*, catches in the Gulf of Thailand: Evidence of transboundary migration of the commercially important fish. Fish. Res..

[2] Koolkalya S., Matchakuea U., Jutagate T. (2017). Growth, Population Dynamics and Optimum Yield of Indian Mackerel, *Rastrelliger kanagurta* (Cuvier, 1816)*,* in the Eastern Gulf of Thailand. Int. J. Agric. Technol..

[3] Food and Agriculture Organization of the United Nations (2021). FAO Yearbook. Fishery and Aquaculture Statistics 2019.

[4] Collette B.B., Nauen C.E. (1983). Scombrids of the World: An Annotated and Illustrated Catalogue of Tunas, Mackerels, Bonitos, and Related Species Known to Date.

[5] Muto N., Alama U.B., Hata H., Guzman A.M.T., Cruz R., Gaje A., Traifalgar R.F.M., Kakioka R., Takeshima H., Motomura H. (2016). Genetic and morphological differences among the three species of the genus *Rastrelliger* (Perciformes: Scombridae). Ichthyol. Res..

[6] Bolger A.M., Lohse M., Usadel B. (2014). Trimmomatic: A flexible trimmer for Illumina sequence data. Bioinformatics.

[7] Marcais G., Kingsford C. (2011). A fast, lock-free approach for efficient parallel counting of occurrences of k-mers. Bioinformatics.

[8] Vurture G.W., Sedlazeck F.J., Nattestad M., Underwood C.J., Fang H., Gurtowski J., Schatz M.C. (2017). GenomeScope: Fast reference-free genome profiling from short reads. Bioinformatics.

[9] Surachat K., Deachamag P., Wonglapsuwan M. (2022). The first de novo genome assembly and sex marker identification of Pluang Chomphu fish (*Tor tambra*) from Southern Thailand. Comput. Struct. Biotechnol. J..

[10] Luo R., Liu B., Xie Y., Li Z., Huang W., Yuan J., He G., Chen Y., Pan Q., Liu Y. (2012). SOAPdenovo2: An empirically improved memory-efficient short-read de novo assembler. Gigascience.

[11] Gurevich A., Saveliev V., Vyahhi N., Tesler G. (2013). QUAST: Quality assessment tool for genome assemblies. Bioinformatics.

[12] Manni M., Berkeley M.R., Seppey M., Zdobnov E.M. (2021). BUSCO: Assessing Genomic Data Quality and Beyond. Curr. Protoc..

[13] Stanke M., Diekhans M., Baertsch R., Haussler D. (2008). Using native and syntenically mapped cDNA alignments to improve de novo gene finding. Bioinformatics.

[14] Huerta-Cepas J., Szklarczyk D., Heller D., Hernandez-Plaza A., Forslund S.K., Cook H., Mende D.R., Letunic I., Rattei T., Jensen L.J. (2019). eggNOG 5.0: A hierarchical, functionally and phylogenetically annotated orthology resource based on 5090 organisms and 2502 viruses. Nucleic Acids Res..

[15] Cantalapiedra C.P., Hernandez-Plaza A., Letunic I., Bork P., Huerta-Cepas J. (2021). eggNOG-mapper v2: Functional Annotation, Orthology Assignments, and Domain Prediction at the Metagenomic Scale. Mol. Biol. Evol..

[16] Camacho C., Coulouris G., Avagyan V., Ma N., Papadopoulos J., Bealer K., Madden T.L. (2009). BLAST plus: Architecture and applications. BMC Bioinform..

[17] Edgar R.C. (2004). MUSCLE: Multiple sequence alignment with high accuracy and high throughput. Nucleic Acids Res..

[18] Stamatakis A. (2006). RAxML-VI-HPC: Maximum likelihood-based phylogenetic analyses with thousands of taxa and mixed models. Bioinformatics.

[19] Beier S., Thiel T., Munch T., Scholz U., Mascher M. (2017). MISA-web: A web server for microsatellite prediction. Bioinformatics.

[20] Meng G., Li Y., Yang C., Liu S. (2019). MitoZ: A toolkit for animal mitochondrial genome assembly, annotation and visualization. Nucleic Acids Res..

[21] Suda A., Nishiki I., Iwasaki Y., Matsuura A., Akita T., Suzuki N., Fujiwara A. (2019). Improvement of the Pacific bluefin tuna (*Thunnus orientalis*) reference genome and development of male-specific DNA markers. Sci. Rep..

[22] Malmstrom M., Matschiner M., Torresen O.K., Jakobsen K.S., Jentoft S. (2017). Whole genome sequencing data and de novo draft assemblies for 66 teleost species. Sci. Data.

[23] Malmstrom M., Matschiner M., Torresen O.K., Star B., Snipen L.G., Hansen T.F., Baalsrud H.T., Nederbragt A.J., Hanel R., Salzburger W. (2016). Evolution of the immune system influences speciation rates in teleost fishes. Nat. Genet..

[24] Barth J.M.I., Damerau M., Matschiner M., Jentoft S., Hanel R. (2017). Genomic Differentiation and Demographic Histories of Atlantic and Indo-Pacific Yellowfin Tuna (*Thunnus albacares*) Populations. Genome. Biol. Evol..

[25] Adelyna M.A.N., Jung H., Chand V., Mather P.B., Azizah M.N.S. (2016). A genome survey sequence (GSS) analysis and microsatellite marker development for Indian mackerel, *Rastrelliger kanagurta*, using Ion Torrent technology. Meta Gene.

[26] Bovine Genome S., Analysis C., Elsik C.G., Tellam R.L., Worley K.C., Gibbs R.A., Muzny D.M., Weinstock G.M., Adelson D.L., Eichler E.E. (2009). The genome sequence of taurine cattle: A window to ruminant biology and evolution. Science.

[27] Marra N.J., Stanhope M.J., Jue N.K., Wang M., Sun Q., Pavinski Bitar P., Richards V.P., Komissarov A., Rayko M., Kliver S. (2019). White shark genome reveals ancient elasmobranch adaptations associated with wound healing and the maintenance of genome stability. Proc. Natl. Acad. Sci. USA.

[28] Steinke D., Hoegg S., Brinkmann H., Meyer A. (2006). Three rounds (1R/2R/3R) of genome duplications and the evolution of the glycolytic pathway in vertebrates. BMC Biol..

[29] Glasauer S.M., Neuhauss S.C. (2014). Whole-genome duplication in teleost fishes and its evolutionary consequences. Mol. Genet. Genom. MGG.

[30] Guo W., Tong J., Yu X., Zhu C., Feng X., Fu B., He S., Zeng F., Wang X., Liu H. (2013). A second generation genetic linkage map for silver carp (*Hypophthalmichehys molitrix*) using microsatellite markers. Aquaculture.

[31] Mastrochirico-Filho V.A., Del Pazo F., Hata M.E., Villanova G.V., Foresti F., Vera M., Martinez P., Porto-Foresti F., Hashimoto D.T. (2019). Assessing Genetic Diversity for a Pre-Breeding Program in *Piaractus mesopotamicus* by SNPs and SSRs. Genes.

[32] Zhang J., Ma W., Wang W., Gui J.-F., Mei J. (2016). Parentage determination of yellow catfish (*Pelteobagrus Fulvidraco*) based on microsatellite DNA markers. Aquac. Int..

[33] Tian H.F., Hu Q.M., Li Z. (2021). Genome-wide identification of simple sequence repeats and development of polymorphic SSR markers in swamp eel (*Monopterus albus*). Sci. Prog..

[34] Jondeung A., Karinthanyakit W. (2010). The complete mitochondrial DNA sequence of the short mackerel (*Rastrelliger brachysoma*), and its phylogenetic position within Scombroidei, Perciformes. Mitochondrial DNA.

[35] Chen Y., Cheng Q., Qiao H., Zhu Y., Chen W. (2013). The complete mitochondrial genome sequence of *Rastrelliger kanagurta* (Perciformes: Scombridae). Mitochondrial DNA.

[36] Iwasaki W., Fukunaga T., Isagozawa R., Yamada K., Maeda Y., Satoh T.P., Sado T., Mabuchi K., Takeshima H., Miya M. (2013). MitoFish and MitoAnnotator: A mitochondrial genome database of fish with an accurate and automatic annotation pipeline. Mol. Biol. Evol..

[37] Catanese G., Manchado M., Infante C. (2010). Evolutionary relatedness of mackerels of the genus Scomber based on complete mitochondrial genomes: Strong support to the recognition of Atlantic *Scomber colias* and Pacific *Scomber japonicus* as distinct species. Gene.

[38] Pang J., Cheng Q., Sun D., Zhang H., Jin S. (2016). The complete mitochondrial genome sequence of *Thunnus alalunga* (Bonnaterre, 1788). Mitochondrial DNA A DNA Mapp. Seq. Anal..

[39] Pang J., Cheng Q., Sun D., Zhang H., Jin S. (2016). The sequence and organization of complete mitochondrial genome of the yellowfin tuna, *Thunnus albacares* (Bonnaterre, 1788). Mitochondrial DNA A DNA Mapp. Seq. Anal..

