# Supplementary figures and images for: The First Genome Survey and De Novo Assembly of the Short Mackerel (Rastrelliger brachysoma) and Indian Mackerel (Rastrelliger kanagurta)
Source: Animals (Basel). 2022 Jul 10;12(14):1769. doi: 10.3390/ani12141769 (PMC9312166; doi:10.3390/ani12141769)

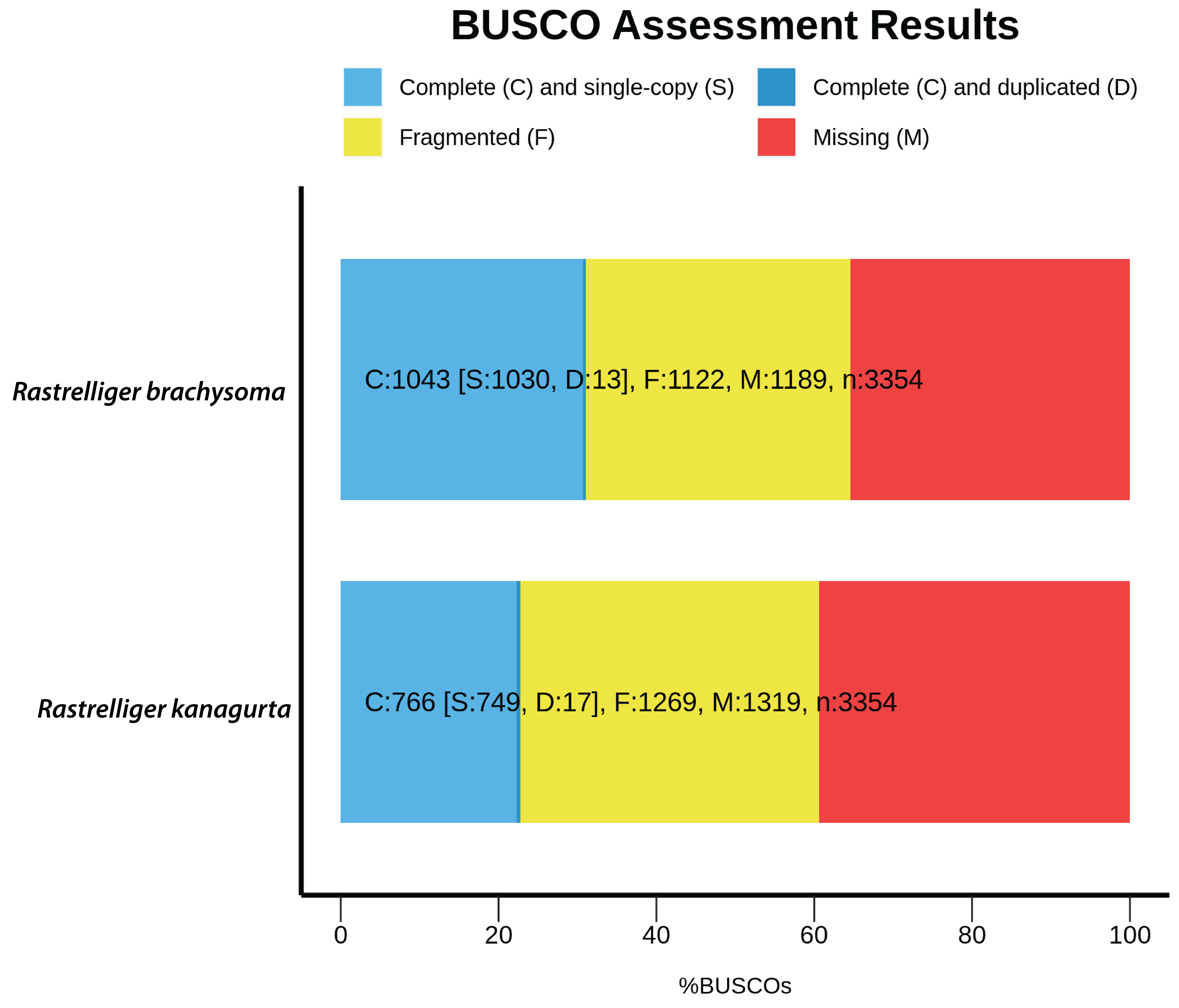

Supplement: Supplementary file 1 [file animals-12-01769-s001.zip › Figure S1.png]
